# A blended intervention to promote physical activity, health and work productivity among office employees using intervention mapping: a study protocol for a cluster-randomized controlled trial

**DOI:** 10.1186/s12889-020-09128-z

**Published:** 2020-06-25

**Authors:** Yan Sun, Aiwei Wang, Siyue Yu, Martin S. Hagger, Xiangyan Chen, Shirley Siu Ming Fong, Chunqing Zhang, Wendy Yajun Huang, Julien S. Baker, Frédéric Dutheil, Yang Gao

**Affiliations:** 1 Department of Sport, Physical Education and Health, Hong Kong Baptist University, Hong Kong, China; 2 JC School of Public Health and Primary Care, The Chinese University of Hong Kong, Hong Kong, China; 3 Psychological Sciences, University of California, Merced, CA USA; 4 Department of Health Technology and Informatics, The Hong Kong Polytechnic University, Hong Kong, China; 5 Department of Health and Physical Education, Education University of Hong Kong, Hong Kong, China; 6 School of Public Health, The University of Hong Kong, Hong Kong, China; 7 LaPSCo, Physiological and Psychosocial Stress, CHU Clermont-Ferrand, Preventive and Occupational Medicine, Witty Fit, University Hospital of Clermont-Ferrand, F-63000 Clermont-Ferrand, France

**Keywords:** Physical activity, Health promotion, Work productivity, Office employees, Blended intervention, Web-based intervention

## Abstract

**Background:**

Regular participation in moderate-to-vigorous physical activity (MVPA) is related to decreased risk of morbidity and mortality. Among working populations, lack of MVPA may also be a risk factor for absenteeism and presenteeism. Both traditional workplace-based and web-based interventions have been suggested as being effective in promoting participation MVPA, health-related outcomes, and work-related productivity. However, several challenges limit their application in real world contexts. A ‘blended’ intervention approach combining the two intervention strategies is proposed to overcome these limitations. The proposed intervention aims to utilize the blended approach to increase participation in MVPA, health-related outcomes, and work productivity among inactive workers.

**Methods:**

The study will comprise of a three-group cluster randomized controlled trial (cluster-RCT), comprising a three-month actual intervention and a nine-month behavioral follow-up period. The three groups will be: a web-based intervention group, a blended intervention group combining the web-based components with face-to-face workshops and posters, and a control group. Physically inactive office employees (*N* = 495) from 33 companies (i.e., clusters) will be recruited and randomly assigned to the three groups by cluster randomization. The intervention mapping (IM) framework will be used for selecting and applying effective health behavioral theories and behavioral change techniques (BCTs) to the development, implementation and assessment of the intervention, which will be personally tailored. The primary outcome variable will be objectively-measured MVPA using an accelerometer. Secondary outcomes will consist of indices of health including adiposity, blood pressure, blood sugar, blood lipids, self-reported depression, anxiety, stress, health-related quality of life and work-related variables including absenteeism and presenteeism.

**Discussion:**

The proposed study adopts a robust blended intervention approach that is expected to overcome challenges in applying workplace-based and web-based interventions separately and yield larger effects in promoting MVPA participation, health-related outcomes and work productivity. Improvements in work productivity outcomes will be of particular interest to employers. If more effective, the new blended intervention has the potential to be implemented on a larger scale to benefit workplace populations.

**Trial registration:**

The trial is prospectively registered at the ClinicalTrials.gov PRS (Trial ID: NCT04391270; Date of First Posted: May 18, 2020).

## Background

Globally, 38 million people die from non-communicable diseases (NCDs) every year, accounting for 68% of all global deaths [1]. Cardiovascular diseases contribute the most to NCD mortality, followed by cancers, chronic respiratory diseases and diabetes. Regular participation in moderate-to-vigorous physical activity (MVPA) is associated with reduced risk of cardiovascular diseases, several cancers, diabetes, obesity, depression, stress and mortality [2]. The World Health Organization (WHO) recommends that adults participate in 150 min of MVPA per week to maintain health and prevent disease. However, near one-quarter of all adults globally do not meet the recommended level of MVPA [3]. In fact, insufficient MVPA is the fourth leading risk factor for mortality and is associated with 3.2 million global deaths each year [2]. Recent evidence has revealed that adults that do not participate in sufficient MVPA are 20 to 30% more likely to die compared to those who participate in sufficient MVPA [3].

In Hong Kong, cancers and cardiovascular diseases are the top two causes of death [4]. However, the latest report from the Hong Kong Department of Health (2016) revealed that an even lower proportion of people are physically active (43.6%, compared to the global level of 75%). Mean values for time spent in moderate- and vigorous-intensity physical activity among Hong Kong residents were 2.1 days/week and 1.3 days/week respectively, whilst its medians were zero for both forms of activity. Another population-based survey revealed that keeping healthy or fit was the strongest facilitator for MVPA (49.2%), whilst lack of time (30.7%), tiredness (17.5%), and laziness (14.6%) were the top three barriers [5]. Compared to children and adolescents, fewer adults were physically active in almost all physical activity parameters, including participation rates, frequency and duration.

Except for health, insufficient MVPA has been suggested to be a potential risk factor for work productivity. Lack of physical activity might reduce work productivity through increased illness-related absenteeism and presenteeism, especially the latter [6, 7]. Presenteeism is not simply the opposite of absenteeism but is a reduced ability to work productively. Though being at work, employees with presenteeism are more likely to reduce work output and make errors. In the U.S., estimated costs related to presenteeism was higher than that for absenteeism (yearly cost: $180 billion vs. $118 billion) [8]. Compared to the strong evidence on the relationship between MVPA and health, the association between MVPA and work productivity is still unclear.

The workplace is an ideal setting to promote MVPA, health and work productivity among working populations. Recent systematic reviews and meta-analyses revealed small-to-moderate effects of interventions for MVPA increases [9]. Interventions based on theories of motivation were suggested to be more effective than other theories, though their effects were still moderate [10]. It has been suggested that a gap between theory and practice may play a role in the relatively small effect observed. Intervention mapping (IM) is a health promotion protocol for selecting and applying effective psychological theories and behavioral change techniques (BCTs) to develop, implement and evaluate health promotion programmes [11]. A recent systematic review found that the IM significantly increased the uptake of disease prevention programmes and recommended it for designing interventions aimed at promoting health [12].

In recent years, web-based interventions have been increasing rapidly due to the popularization of internet use, which has been considered to be more effective and cost-effective than traditional workplace-based interventions, which included face-to-face workshops, counselling, posters and printed materials [9]. However, web-based interventions usually had very low participant engagement and retention rates, mainly due to lack of interactions with individuals and a limited ability to involve verbal, aural and physical cues [13]. Furthermore, long-term effects (≥ 3 months) were also compromised as quite a large portion of users lost interest [14]. A blended approach combining web-based and traditional workplace-based interventions might be able to mitigate these limitations while maintaining distinct advantages [13]. However, few interventions adopted this approach and most of them are still ongoing. In addition, methodologies across those studies varied largely, particularly in relation to the workplace-based components involved. There is only one study comparing a blended approach (web-based + center-based) to a purely web-based approach to increase MVPA among adults 50 years of age and above [14]. Results from that study favored the blended over the web-based only means (effect size: 0.20 vs. 0.06). Thus, it is unclear if the blended interventions are more beneficial than web-based interventions for the working population to promote MVPA.

In Hong Kong, more adults are insufficiently active than children, and while great efforts have been created to improve MVPA levels among children, little attention has been paid to working adults. There is an urgent call for effective interventions to increase MVPA, health, and work productivity in this population. After a comprehensive review of existing evidence, the current blended intervention using IM was therefore conceived.

### Study objectives

The primary objective of this study is to examine between-group differences in changes of objectively measured MVPA levels (min/week) among a randomly selected sample of office employees who are physically inactive in Hong Kong. Secondarily, we will also address the following objectives:

- To examine between-group differences in changes of self-reported MVPA levels (min/week) in different domains among the sample;
- To examine between-group differences in changes of objectively measured physical health-related outcomes (including body mass index (BMI), percentage of body fat (%BF), waist circumference, blood pressure and fasting blood sugar and blood lipids) among the sample;
- To examine between-group differences in changes of self-reported mental health-related outcomes (including depression, anxiety and stress) and health-related quality of life among the sample;
- To examine between-group differences in changes of work-related outcomes (including absenteeism and presenteeism) among the sample;
- To examine between-group differences in participants’ engagement and retention among the sample;
- To examine mediation and moderation effects of the theoretical determinants of MVPA increase (such as motivation, self-efficacy and intention) and socio-demographic characteristics among the sample.

## Methods and design

### Study design

The study will be a double-blind 3-group cluster-RCT developed following the IM protocol, lasting for 12 months (3-month intervention + 9-month follow-up). The target population will be physically inactive office employees (< 150 min/week of MVPA) in Hong Kong. With the workplace as the cluster unit for randomization and intervention, participants will be randomly recruited and assigned into the following three groups: web-based intervention group, blended intervention group (combining web-based intervention content with face-to-face workshops and environmental changes at the workplace), and a control group. A website will be developed for this study, consisting of two main sub-sections: “Library” (providing general information of MVPA and health) and “Intervention” (providing personally-tailored theory-based intervention). All participants will have access to “Library”, whilst only those in the two intervention groups can visit the “Intervention”. In addition, the participants in the blended group will also receive three workshops and environmental changes at their workplaces. This intervention study was developed following the IM protocol and mainly based on social psychological theories of intentional behaviour and motivation. In particular, it was based mainly on changing beliefs relating to MVPA from the theory of planned behaviour (TPB), along with effective behavioural change techniques (BCTs) aimed at changing motivation, self-regulation skills and habit-formation. The primary outcome variable will be changes in MVPA. Secondary outcomes will consist of health-related indices (including body weight, central adiposity, blood pressure, blood sugar, blood lipids, depression, anxiety, stress and quality of life), work-related indices (including absenteeism and presentism) and theory-related determinants (such as intrinsic motivation, self-efficacy, and intention). Evaluations will be conducted at pre-intervention (T1), post-intervention (T2), as well as 9 months following the intervention (T3). Both the participants and outcome assessors will be blinded from the assignment results to the intervention. This study has received support from the General Research Fund, University Grants Committee of Hong Kong (Ref. No.: 12609919).

### Study population

The target population will be physically inactive office employees in Hong Kong. A cluster random sampling method will be adopted to recruit the participants. Firstly, our research team members will randomly select study companies from the latest company list obtained from the Census and Statistics Department of Hong Kong (CSD), and then randomly and equally assign them into the three study groups. Randomization will be performed using the random function available in Excel. The CSD maintains a computerised Central Register of more than 40-million active companies in Hong Kong and updates the information on a quarterly basis. A company will be excluded if: (1) the number of employees is less than 100; and, (2) there are other ongoing programmes involving MVPA promotion in that company, due to the following considerations: (1) given the insufficient MVPA prevalence of 55% among the local adults and low participation rates reported from previous studies [15], it will largely increase workload and decrease feasibility of the study if the employee number of a company is too small, particularly in relation to outcome measurements and workshop delivery at the workplace. In addition, the CSD uses the employee numbers of 10, 50, and 100 as cut-off points to classify companies, in which 100 is best suitable for our study; and, (2) such activity programmes will bias our results. In addition, industry sections and work settings will be considered in company selection and assignment. Secondly, all employees in a recruited company (or a workplace if the company has more than one workplace addresses) will be invited to participate in the study. The short version of the International Physical Activity Questionnaire (IPAQ short version) will be employed to screen for eligibility [16]. Only those who do NOT meet the WHO’s recommended MVPA levels (MVPA < 150 min/week) will be eligible and then recruited in the study. For safety, the Physical Activity Readiness Questionnaire (PAR-Q) will also be completed by the employees. Those with “Yes” to one or more questions will need to seek a doctor’s approval before participation. In addition, employees will be excluded from the study if they report any conditions preventing them from being active. The written informed consent form will be collected from each participant. The study has received ethics approval from the Research Ethics Committee (REC) of Hong Kong Baptist University (Ref. No.: HASC/17–18/0529).

Sample size estimation: existing evidence suggested small-to-moderate effects of interventions for MVPA increase. An effect size of 0.40 was adopted from a recent study, as it was considered similar to our study (e.g., Asian population, web-based, using IM to develop the intervention) [17]. We decided on a cluster size of 15 after a full consideration of several factors, such as suitable participant number to a workshop, prevalence rate of insufficient MVPA, participation rate and company size. Thus, using an ICC of 0.01, α of 0.05, β of 0.8, and an attrition rate of 30%, a total of 465 participants in 31 clusters was estimated (each group: 155 participants in 10.3 clusters) [15, 18]. We further rounded up the cluster number in each group to 11 (consisting of 165 participants), finally yielding a cluster number of 33 and a participant number of 495 for this intervention.

### Intervention mapping (IM)

The intervention is developed following the IM. It is an intervention protocol for selecting and applying effective health behavioural theories and BCTs to develop, implement and assess the intervention programmes [11, 19]. It helps to bridge gaps between theory and practice. Evidence has suggested that it may increase effectiveness and uptake of interventions [11, 12]. There are six steps in the IM framework and each consists of specific tasks and depends on findings of the preceding step. Step 1 is a needs assessment based on the PRECEDE model. As suggested, we have conducted a comprehensive literature review on the problem size of insufficient MVPA, its impact on health and work productivity, and facilitators and barriers of being active among Hong Kong adults, by searching for relevant journal articles, governmental reports and statistics and PhD dissertations. We will perform two gender-specific focus groups among a convenient sample of inactive office employees and two in-depth interviews with company managers to further specify key modifiable individual and environmental determinants of MVPA and possible ways to promote MVPA. Information collected in Step 1 which will guide us to develop SMART intervention objectives in Step 2. In addition, we will form a steering group, consisting of diverse relevant stakeholders, including research team members, managers and office employees. Our research team consists of experts in medicine, public health, psychology and sports science. In Step 2, we will clarify expected changes in both behaviours and environments, and define performance objectives and their theoretical determinants. For example, if an expected change of behaviour is to increase MVPA at home, examples of performance objectives may be to (1) develop an intention to be active at home and (2) make a plan to be active at home. For a performance objective of developing the intension to be active at home, the theoretical determinants may include attitudes, self-efficacy and subjective norms [20]. Step 3 is a selection of theory-based methods and practical strategies, in which we have reviewed relevant intervention studies and identified effective BCTs for MVPA change, as shown in Table 1 [22]. We will further revise those methods and strategies according to findings from the focus groups and in-depth interviews described in Step 1. In the 4th step of programme production, we will consult with all stakeholders to refine the intervention objectives and strategies, develop intervention materials and then conduct a pilot study to test and revise the strategies and materials. Implementation feasibility and resource constraints will be given full consideration in this step. Upon completion of the first 4 steps, a web-based platform will then be developed. In Step 5, a plan for how the intervention will be implemented is constructed. In the blended intervention group, we have decided to choose an employee from each participating workplace to help deliver the face-to-face workshops as a facilitator [20]. An operational manual and training materials will be developed. The involvement of an employee can provide a source of informal peer leader influence and ensure the sustainability of the intervention, as no extra human resource outside the workplace is needed. In Step 6 (the last step), we have developed a plan to evaluate effectiveness and process of the intervention based on the literature review, which will be finalised according to the preceding steps.

**Table 1:** Timeline, questions, personally-tailored feedback, and BCTs involved in six web-based sessions in two intervention groups^a^

Session/ *Time*	Questions asked	Personally-tailored feedback/ *Theoretical strategy involved*	BCTs involved/ Name *(code)*^b^
Session 1 *(Week 1)*	• MVPA in the past week • MVPA goal • MVPA self-efficacy • Preferred MVPA routine • Habit strength of MVPA	• MVPA guideline related to the main motivation selected *(motivational strategy)* • Feedback on current MVPA level related to the main motivation *(self-regulation strategy)* • Setting a goal for the next 2 weeks based on self-efficacy level *(self-regulation strategy)* • Developing habits based on preferred activity *(habit development strategy)* • Recognising prompts related to habit strength level *(habit development strategy)*	• Goal setting (behaviour)*(1.1)* • Feedback on behaviour *(2.2)* • Information about health consequences *(5.1)* • Information about emotional consequences *(5.6)* • Prompts/cues *(7.1)* • Habit formation *(8.3)* • Verbal persuasion about Capability *(15.1)*
Session 2 *(Week 3)*	• MVPA in the past week • Weight^c^ • Goal setting • MVPA goals (both long- and short-term, open questions) • Habits and prompts • Action planning phrased with/without reference to habit (open answers)	• MVPA guidance refresher based on the main goal *(self-regulation strategy)* • MVPA progress feedback • Long- and short- term SMART goals *(motivational strategy)* • Feedback on developing habits and noticing prompts *(habit development strategy)* • Action plan *(self-regulation strategy)* • Action plan with reference to behaviour repetition in a stable context *(habit development strategy)*	• Goal setting (behaviour)*(1.1)* • Goal setting (outcome) *(1.3)* • Action planning *(1.4)* • Feedback on behaviour *(2.2)* • Prompts/cues *(7.1)* • Habit formation *(8.3)* • Graded tasks *(8.7)*
Session 3 *(Week 5)*	• MVPA in the past week • Weight • Coping self-efficacy • Action plan completed • Main prompts • Action plan evaluated (open answers) • Confidence to recognise prompts	• Boosting confidence and staying motivated based on identified barriers *(motivational strategy)* • MVPA progress feedback including graphs *(self-regulation strategy)* • Feedback on developing habits *(habit development strategy)* • Noticing prompts *(habit development strategy)* • Action plan *(self-regulation strategy)* • Action plan with reference to behaviour repetition in a stable context *(habit development strategy)*	• Action planning *(1.4)* • Review behaviour goal(s) *(1.5)* • Discrepancy between current behaviour and goal *(1.6)* • Monitoring of emotional consequences *(5.4)* • Anticipated regret *(5.5)* • Information about emotional consequences *(5.6)* • Prompts/cues *(7.1)* • Habit formation *(8.3)*
Session 4 *(Week 7)*	• MVPA in the past week • Weight • Positive social support • Negative social support • Influence of others	• MVPA progress feedback *(self-regulation strategy)* • Positive influence of others and dyadic plans *(motivational strategy)* • Positive influence of others and dyadic plans *(self-regulation strategy)* • Positive influence of others and dyadic routines *(habit development strategy)* • Negative influence of others and staying motivated when others are not supportive • Encouraging others to be active	• Review behaviour goal(s) *(1.5)* • Discrepancy between current behaviour and goal *(1.6)* • Review outcome goal(s) *(1.7)* • Social support (practical) *(3.2)* • Social support (emotional) *(3.3)* • Information about others’ approval *(6.3)* • Self-reward *(10.9)* • Restructuring the social environment *(12.2)*
Session 5 *(Week 9)*	• MVPA in the past week • Weight • Behavioural barriers • Re-evaluating short-term goal for the intervention (open questions) • Habit development	• MVPA progress feedback *(self-regulation strategy)* • Maintaining positive habits – coping planning *(habit development strategy)* • Relapse prevention based on the main barrier selected with an emphasis on staying motivated *(motivational strategy)* • Relapse prevention based on the main barrier selected with an emphasis on staying motivated and self-regulating *(self-regulation strategy)* • Relapse prevention based on the main barrier selected with an emphasis on staying motivated/self-regulating and maintaining positive habits *(habit development strategy)*	• Problem solving *(1.2)* • Review outcome goal(s) *(1.7)* • Reduce prompts/cues *(7.3)* • Behaviour substitution *(8.2)* • Habit reversal *(8.4)* • Reducing negative emotions *(11.2)* • Avoidance/ reducing exposure to cues for the behaviour *(12.3)*
Session 6 *(Week 11)*	• MVPA in the past week • Weight • Habit strength	• MVPA progress feedback with graph *(self-regulation strategy)* • Weight changes throughout the program with graph *(self-regulation strategy)* • Tips to stay motivated *(motivational strategy)* • Tips to stay motivated and to self-regulate MVPA *(self-regulation strategy)* • Tips to stay motivated and to self-regulate MVPA and to follow newly developed routines *(habit development strategy)* • Success stories from other participants who increased MVPA levels *(motivational strategy)*	• Feedback on behaviour *(2.2)* • Feedback on outcome(s) of behaviour *(2.7)* • Information about health consequences *(5.1)* • Information about emotional consequences *(5.6)* • Information about others’ approval *(6.3)* • Behavioural practice/rehearsal *(8.1)* • Pros and cons *(9.2)*

*Abbreviations*: *BCTs* behavioural change techniques, *MVPA* moderate-to-vigorous physical activity

^a^ The table was modified from [10] and will be reviewed according to results of focus groups and in-depth interviews

b Names and codes of BCTs were drawn from [21]

c Weight is an example of selected health effects from increased MVPA

### Intervention content

#### Control group

The participants in the control group will have access to the “Library” sub-section of the website. General information of MVPA, health and work productivity will be provided with 18 short essays (1–2 A4 pages in length), which can be downloaded and printed. The information will be factual and non-personally tailored. Some examples of topics are the recommended MVPA levels, benefits of being active and consequences of being inactive. Three BCTs will be applied in this group, including information about health consequences, social and environmental consequences and emotional consequences (Codes of 5.1, 5.3 and 5.6) [22]. Every 2 weeks in three consecutive months, we will upload three essays on the “Library” and the participants will receive a reminder to visit the website (Table 1). The participants will be asked to evaluate each essay and each session on usefulness and acceptability (as described in “*Process evaluation*” sub-section). Upon completion of the whole study, the participants in the control group will be allowed to access the “Intervention” sub-section of the website to receive the web-based intervention, as outlined next.

#### Web-based intervention group

Except “Library”, the participants in this group will also have access to the “Intervention” sub-section of the website. It will consist of six sessions delivered every 2 weeks following the same time schedule as the essay delivery in “Library” (Table 1). In each session, the participants will be asked about their MVPA, related theoretical determinants, related social and environmental factors. Then, they will receive personally-tailored feedback incorporating relevant BCTs aiming to promote motivation, self-regulation and habit development [10, 22]. If-then algorithms will be used in this platform. That is, the feedback will be provided based on individual response to the questions. A total of 29 BCTs have been selected by our research team, which will be further refined according to the results from the first two steps of the IM protocol (Table 1). For example, the participants will be advised on how to comply towards their goals and maintain motivation in face of barriers (motivational strategy); they will be encouraged to set outcome-specific goals and self-monitor their progress towards the goals by monitoring how much time they spent in MVPA (self-regulation strategy); and, they will be asked to recognise and identify situational, contextual and time-based cues that can prompt them to be active (habit development strategy). Since the second session, the participants will also receive a recap of the messages from the previous session before the new content and feedback. Pictures and graphs will be used along with text where if it is applicable (e.g., graphs to show MVPA changes during the intervention).

#### Blended intervention group

Except for visiting “Library” and “Intervention” of the website, the participants in this group will also receive three face-to-face workshops (40 min per session) held at their workplaces during lunchtime in Weeks 2, 4 and 8. We decided on a shorter time interval between the first two sessions, as the participants may face more problems and need more encouragement and support at the beginning of the intervention. The workshops will mainly aim at solving problems that they have faced using the website, sharing experiences with each other and encouraging the participants to adhere to the intervention [13]. Four more BCTs will be added in, including *Problem solving (1.2), Instruction on how to perform the behaviour (4.1), Re-attribution (4.3)* and *Mental rehearsal of successful performance (15.2)* [22]. Taking an example of *mental rehearsal of successful performance*, the participants will be asked to imagine walking after dinner at home. Mental rehearsal has been proven to increase changes in several health behaviours [23]. An employee from each participating workplace will be selected to help deliver the workshops as a facilitator [20]. In addition, further strategies of environmental changes at the workplace will also be adopted in this group, including posters (e.g. point-of-choice prompts of stair climbing, health benefits of being active) and management support letters [20].

### Outcome evaluation of the intervention

Outcome evaluations will be performed three times at pre-intervention (T1), post-intervention (T2), and 9 months after intervention (T3).

#### Primary outcome variable

MVPA will be objectively measured by a hip-worn accelerometer (ActiGraph, Pensacola, FL, Models GT3X or GT3X+) at T1-T3. The participants will be instructed to wear an accelerometer on the right hip during waking hours for seven consecutive days, except when bathing, performing water-based activities and sleeping [24]. In this study, 1-min epochs will be selected to record data [25]. The data from the accelerometers will be rectified, integrated and then stored as activity counts per minute, representing the intensity of the activity performed. Non-wear time is defined as 60 or more consecutive minutes of zero counts [26]. Freedson’s cut-off points will be used to classify vigorous-intensity PA (VPA), moderate-intensity PA (MPA), and light-intensity PA (LPA) [27]. Data of at least 10 h per day for a minimum of 4 days, consisting of at least three weekdays and one weekend, will be regarded as valid and included in data analysis [28].

#### Secondary outcome variables

Physical health-related outcomes: Height will be measured to the nearest 0.1 cm using a stadiometer (Holtain Ltd., Pembrokeshire, United Kingdom). Weight will be measured to the nearest 0.1 kg on a calibrated digital balance scale (Seca, max. 200 kg, Germany) with the participants wearing lightweight clothing and no shoes. Body mass index (BMI, kg/m^2^) will be calculated from weight and height. Waist circumference (cm) will be measured midway between the lowest rib margin and the top of the iliac crest at the end of gentle expiration [29]. The measure will be performed with a flexible meter ribbon accurate to 0.1 cm. %BF will be assessed by a Tanita TBF-410 Body Composition Analyzer (Tanita Corporation, Tokyo, Japan) using foot-to-foot bioelectrical impedance analysis (BIA). Blood pressure will be measured using the Omron M6 Compact (HEM-7000-E, Omron Healthcare Corporation, Kyoto, Japan) following standard measurement protocols [30]. Fasting blood glucose, triglycerides (TG) and high-density lipoprotein (HDL) will be measured using the Reflotron® Plus system (Roche Diagnostics, F. Hoffmann-La Roche Ltd., Burgess Hill, UK) [31]. Following a 10-h overnight fast, a total of 100 μl fingertip blood samples will be collected by a registered research nurse using a heparinized capillary tube (Microvette®100) and immediately pipetted onto three test strips (32 μl each) for blood glucose, TG and HDL analyses respectively.

Mental health-related outcomes: Depression, anxiety and stress will be measured using the Chinese version of the Depression Anxiety Stress Scale (DASS-21) [32, 33]. It is a widely used self-administered physiological instrument with sound validity and reliability. It consists of 21 items in three domains (including depression, anxiety, and stress) on a 4-point Likert scale (ranging from 0 (did not apply to me at all) to 3 (applied to me very much or most of the time)). Total scores in each domain will classify the participants into “normal”, “mild”, and “moderate and above” groups, according to the DASS manual [32].

Health-related quality of life will be measured using the Chinese version of the 12-item Short-Form Health Survey Version 2. It is a popular health-related quality of life measure, including physical and mental health domains. The Chinese version has been tested to be valid, reliable and sensitive for Hong Kong populations [34].

Work-related outcomes: Absenteeism records will be collected from each study company, including those in the past 3 months before the study and during the entire study period [10]. Presenteeism refers to reduced work productivity resulting from mental and physical conditions, despite being present at the workplace. It will be assessed using presenteeism questions derived from the WHO Health and Work Performance Questionnaire (HPQ) [7]. It consists of three items on an 11-point Likert scale, ranging from 0 (worst job performance) to 10 (top performance) and has been suggested to be most suitable for PA research, especially for detecting changes in intervention studies [6].

Theory-related determinants of interest will be measured using several valid and reliable psychometric instruments adapted to make references to MVPA [10], including attitudes (4 items), outcome expectations (6 items), subjective norms (2 items), perceived behavioural control (2 items), self-efficacy (6 items), barriers self-efficacy (5 items), intentions (3 items), action planning and coping planning (4 items) and goal facilitation and goal conflict (2 times). In addition, the habit strength of MVPA will be assessed with the Self-Report Behavioral Automaticity Index (8 items) [35].

### Process evaluation of the intervention

Process evaluation is used to monitor and document intervention implementation, and aid in understanding the relationship between specific intervention components and intervention outcomes. A framework model for designing process evaluation of cluster RCTs will be adopted [36]. A fidelity protocol will be developed based on a conceptual framework for implementation fidelity [37].

### Other measures

Socio-demographic characteristics will be collected at baseline as potential confounders, including gender, age, birthplace, resident status, the residential period in Hong Kong, marital status, education, work experience, current job position, years of work (in total and at current job position), current working hours and days in a typical week, current type of shift work, district of residence, type of housing, family size, personal salary and family income [38]. In addition, common chronic diseases (diagnosed by a doctor) will also be collected at baseline.

### Procedure

**Fig. 1:**
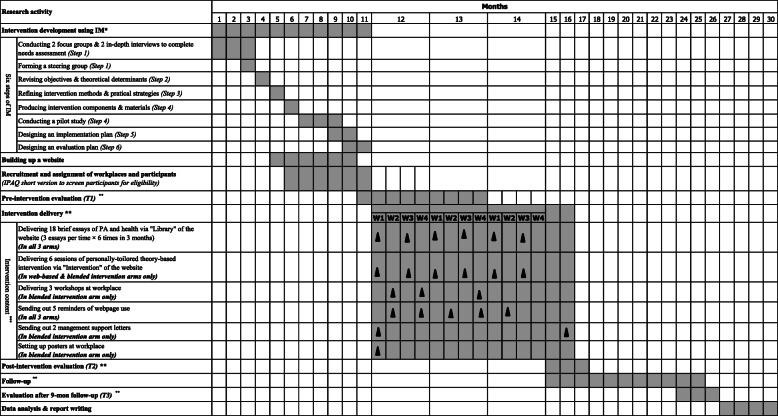
Gantt chart of research activities. *: IM: Intervention mapping. **: Lengths of outcome evaluations at each time point, intervention and follow-up will be 1 month, 3 months and 9 months respectively. As the participant recruitment will last for 3 months, 2 more months were therefore estimated in each activity. ***: Time schedule of intervention delivery in this figure is suitable for participants recruited in the 11th month only. Time schedules for those recruited in Months 12 & 13 will be extended correspondingly

Figure 1 illustrates the procedure of intervention development, participant recruitment, data collection and implementation of the intervention. In brief: (1) focus groups and in-depth interviews will be conducted and a steering group will be formed for the development of the intervention according to the IM framework; (2) participating companies will be randomly selected and assigned into the three study groups; (3) all employees will be invited into the study and be screened for eligibility for participation using an online questionnaire consisting of the IPAQ short version; (4) all participants will receive the outcome evaluations (both primary and secondary) within 1 month before the implementation of the intervention (T1). They will also receive the link to the website and a personalized login/password; (5) during the three consecutive months of intervention, they will receive a reminder sent every 2 weeks via email or WhatsApp to encourage them to use and interact with the website (Weeks 1, 3, 5, 7, 9, and 11). The participants in the control group can only access the “Library”, whilst those in the other two groups can access both the “Library” and “Intervention” sub-sections of the website. A further reminder will be sent to those who do not visit the website 1 week after (Weeks 2, 4, 6, 8, and 10). In addition, the participants in the blended intervention group will also receive three face-to-face workshops (Weeks 2, 4, and 8), management support letters and posters at the workplace. The process evaluation will be conducted for each session and the overall intervention during the intervention; (6) two more outcome evaluations will be performed within 1 month immediately after the intervention and 9 months later (T2 and T3); (7) The “Intervention” sub-section of the website will be open to the participants in the control group after completion of the last wave of evaluations (after T3).

### Data analysis

Continuous variables (e.g., MVPA levels, min/week) will be presented with mean (standard deviation, SD) and/or median (interquartile range, IQR). All continuous variables at T1 will be tested for between-group differences using one-way Analysis of Variance (ANOVA). Normality will be checked by QQ-plots and tested for equal variance assessed by Levene’s test. If skewed, the data will be logarithmically transformed before applying one-way ANOVA. A generalized linear mixed model (GLMM) will be used to compare between-group differences in MVPA changes at T2 and T3, where group and time are the two factors of interest. Time points will be nested with individuals who will be nested within the workplaces. Both individuals and workplaces will be set as random effects. Linear regression and generalised linear models will be used to analyse moderating effects (e.g. theoretical determinants) and mediating effects (e.g. socio-demographic variables) of intervention effectiveness [39]. A significance level of 0.05 will be adopted (two-tailed test). Intention-to-treat analysis will be adopted and performed. Missing data will be imputed using multiple imputations with chained equations, except for those from drop-outs, which will be imputed with the baseline data [10].

### Oversight and monitoring

Data monitoring committee (DMC) is not needed because this trial: 1) has a short duration (i.e., three months); 2) is with known risks that are minimal;, and 3) is on behavioral issues.

Potential adverse effects of intervention: It is critical that measures of harm or unintended consequences are included in interventions targeting PA, to ensure that the interventions are safe and appropriate [40]. Possible adverse effects (e.g. sports injury) suggested by previous studies will be monitored closely and remedied promptly. In addition, diverse means will be undertaken to prevent these consequences in the study (e.g., to inform participants of the possible adverse effects in advance and provide feasible and practical preventive strategies to them).

Important protocol modifications (e.g., changes to eligibility criteria, objectives, analyses) will require a formal amendment to the protocol. Such amendment will be documented in-process and final reports to the funding body and agreed upon by the REC if there is a need (e.g. changes to eligibility criteria) prior to implementation.

### Participant recruitment and retention strategies

Several effective strategies for participant recruitment and retention suggested by previous studies will be adopted in this study, including emphasizing study benefits to participants and the community (e.g. expected health benefits, free physical check-up such as blood glucose test), providing financial incentives and awards for completing all measurements of outcomes (a HK$50 coupon for each participant who completes the measurements at each wave (T1-T3) and a HK$1000 award to five participants who complete all measurements of all waves), keeping the steering group well-functioning, providing meals in the workshop sessions, and so forth [35]. The strategies will be discussed with all stakeholders who will be involved during the intervention development stage, including steering group members and participants of the focus groups, in-depth interviews and pilot study. Amendments will be made if necessary.

### Pilot study

A 3-month pilot of the blended intervention will be conducted to test the participant recruitment and maintenance, data collection instruments and observe the adaptability of the intervention. Participants will be recruited from different work settings with consideration of the characteristics of the companies, with each group consisting of 8–10 participants. Amendments will be made where necessary. The participants in the pilot will not be involved in the main study.

### Time schedule of the study

The proposed study will be completed in 30 months from 01 Jul 2020 to 30 Dec 2022, as shown in Fig. 1.

## Discussion

Being physically active has been related to reduced risks for several major NCDs and morbidity [2]. It might also play a role in increasing productivity at work [6, 7]. More than half of the adults in Hong Kong lack sufficient MVPA to maintain their health and prevent diseases [41]. It is urgent for effective interventions to promote their MVPA. Traditional workplace-based interventions have never been adopted by researchers and policy-makers to address this issue. This type of interventions usually undertakes a comprehensive approach, consisting of intervention means to change individual behaviors’ (e.g. workshop and counselling), organizational culture (e.g. organizational rules), and workplace environment (e.g. posters and facilities) [9, 42]. However, the number of workplace-based interventions has been decreasing in recent years due to the emergent of web-based interventions. Along with a rapid increase in internet and smartphone use, the development and dissemination of web-based interventions seem to be more valuable and worthwhile [43]. For example, web-based interventions can reach a large scale of the population; participants can access flexibility in time and place for participation; interventions can be personally tailored to suit both family and working circumstances; and more support and advice can be obtained outside the workplace [13, 43]. However, low engagement and retention of participants have limited the implications of web-based interventions. For example, a study examining engagement and nonusage attrition among 16,948 users of the 10,000 Steps Australia programme indicated a mean for overall usage days of 34.5 (ranged from 1 to 296 days, SD: 30.5 days) [44]. Among a subsample of 11,651 users who used the platform for at least 3 months, only 50% of them remained using the programme after 30 days. Such figures further decreased to 25% after 42 days [44]. Furthermore, the weekly duration of usage also decreased along with the increasing length of membership, from a mean of 4.1 days/week (SD: 2.4) in the membership length of 1–2 months going down to 1.1 days/week (SD: 0.9) in that of 9–10 months, elucidating that longer duration of usage produced poorer engagement and retention [44]. Similar patterns were also observed in other web-based interventions [16, 45, 46]. Thus, though most web-based PA interventions reported effective, considering the low engagement and retention of participants, the effect size in the real world would be small [45]. More recently, blended interventions have emerged, which combine web-based and traditional workplace-based approaches and might be more effective than each individual approach [13]. For example, with regard to cost-effectiveness and time-effectiveness, blended interventions may decrease the chances of face-to-face communications, resulting in reducing the costs and saving counselling as well as travel time [13]. On the other hand, traditional face-to-face communications can also increase engagement and retention of participants [13]. Probably, the majority of employees who are equipped with computer and internet skills are the optimal target population, adding conventional workplace-based interventions might boost intervention effectiveness. Therefore, we adopted the blended approach in this study to examine if the blended intervention would yield larger effects than the web-based intervention. As far as we know, very few studies adopted the new blended approach to improve MVPA levels for employees and none of them involved assessment of work productivity as suggested in this study.

The proposed intervention would yield impacts on both research and society. Academically, it would add knowledge in research on how to effectively improve MVPA, health and productivity among employees. We will share our experiences with other researchers via diverse channels, including publishing papers in international peer-reviewed journals, delivering presentations on international conferences and sharing the website publicly. If the results are in favor of the blended approach over the web-based method, the new emerging intervention will serve as an example of use for other researchers in this field. In addition, it would inspire researchers to tackle other health issues and address other populations by modifying the new intervention protocol. In terms of its contribution to the society, there would be 330 participants from the 22 companies in the two intervention groups benefiting from the study. Then, we will deliver the blended intervention to those in the control group (165 participants in 11 companies) to improve their MVPA, health and productivity levels. Given its nature of relatively low cost, easy-to-operate and no expert involved in the implementation of the intervention, we expect that the whole product of the blended intervention would be helpful for all the 33 participating companies to independently promote their employees’ MVPA, health and work productivity after completion of the study. In addition, we will propose and assist health governments to advocate and disseminate the blended intervention among all companies to tackle insufficient PA and further improve health status and work productivity among the entire working population of Hong Kong. Other countries and regions with similar demographics to Hong Kong can also learn and benefit from our product.

This study is strengthened by its 3-group cluster-RCT design using the IM framework. The major strengths of using the IM protocol include the systematic literature review and consideration of changes in both the behaviors and environments from theoretical determinants to practical strategies [47, 48], which makes the IM approach much superior than any other methods for conducting this blended intervention study. In addition, the blended pattern would maximize the advantages of both the workplace-based and web-based interventions and therefore has the potential for achieving larger effects than each individual intervention [13]. Furthermore, the IM fully considers the needs of large-scale stakeholders including employees, local companies, health governments, potential facilitators and researchers [48]. Despite its strengths, the IM approach is definitely a time-consuming process. The systematic literature review, defining performance objectives and theoretical determinants, selecting optimal strategies and methods based on both theories and practice and considering back and forth to implement and evaluate the intervention, all would require time. However, the time commitment would be well worth the effort when disseminating the more effective blended intervention to masses of working populations in the future [47].

## Data Availability

Not applicable.

## Abbreviations

ANOVA: Analysis Of Variance
BCTs: Behavioural Change Techniques
BIA: Bioelectrical Impedance Analysis
BMI: Body Mass Index
CSD: Census and Statistics Department
DASS: Depression Anxiety Stress Scale
DMC: Data Monitoring Committee
GLMM: Generalized Linear Mixed Model
HDL: High-Density Lipoprotein
HPQ: Health and Work Performance Questionnaire
IM: Intervention Mapping
IPAQ: International Physical Activity Questionnaire
IQR: Interquartile Range
MVPA: Moderate-to-Vigorous Intensity Physical Activity
NCDs: Non-Communicable Diseases
RCT: Randomized Controlled Trial
REC: Research Ethics Committee
SD: Standard Deviation
TG: Triglycerides
TPB: Theory of Planned Behaviour
WHO: World Health Organization
